# Knowledge Exchanges and Decision-Making Within Hospital Dementia Care Triads: An Ethnographic Study

**DOI:** 10.1093/geront/gnaa216

**Published:** 2021-02-18

**Authors:** Rachael Kelley, Mary Godfrey, John Young

**Affiliations:** 1 Centre for Dementia Research, School of Health and Community Studies, Leeds Beckett University, Leeds, West Yorkshire, UK; 2 Leeds Institute of Health Sciences, School of Medicine, University of Leeds, Leeds, West Yorkshire, UK

**Keywords:** Decision-making, Dementia, Ethnography, Family caregivers, General hospitals

## Abstract

**Background and Objectives:**

Important decisions about the future care of people living with dementia are routinely made in hospitals. Very little is known about how the care needs of hospitalized people with dementia are understood, or how the perspectives of the person, families, and staff intersect to inform decision-making. This study explores how the care needs of people with dementia are understood by the person, their family, and hospital staff (the care triad), and how these perspectives shape decision-making.

**Research Design and Methods:**

Ethnographic data were collected from 2 care-of-older-people general hospital wards via observations, conversations, and interviews with people with dementia, families, and staff. In total, 400 hr of observation and 46 interviews were conducted across two 7- to 9-month periods.

**Results:**

The person’s care needs were often understood differently between and within arms of the care triad. A lack of consistent engagement with families and people with dementia reduced opportunities to recognize and integrate this range of views, leading to delays or difficulties in decision-making. People with dementia, particularly those lacking capacity, were most likely to have their perspectives overlooked.

**Discussion and Implications:**

Early engagement with people with dementia and their families is required to ensure that all perspectives on the person’s current and future care needs are understood and represented during decision-making. Particular attention should be paid to involving people living with dementia in discussions and decisions about their care, and to the assessment and involvement of people who may lack capacity.

Dementia is a global health problem, with 46.8 million people currently living with dementia (Prince et al., 2015). These high rates are reflected in the considerable numbers of people with dementia admitted to general hospitals. In the UK, for example, a quarter of hospital patients have dementia (Sampson et al., 2009). The care needs of hospitalized people with dementia vary greatly, influenced by the degree and effects of their cognitive impairment and physical ill health, encompassing a wide range of comorbid medical problems (Bunn et al., 2014). These include conditions which may exacerbate their dementia (such as delirium or infections), creating a complex and highly varied set of care needs.

This complexity creates challenges for assessing and making decisions about each person’s current and future care needs. In addition, the presence of dementia creates unique challenges for involving people in understanding and making decisions about their care. Each person’s ability to make an informed decision, and so the extent to which input from families or staff is required, will vary depending on the nature of the decision and the stage or effects of their cognitive impairment (Pecanac et al., 2018; Wolfs et al., 2012). Research in other settings indicates that many people with dementia have the ability and desire to participate in decision-making (Daly et al., 2018; Fetherstonhaugh et al., 2013), yet there is very limited understanding of how decision-making happens in practice for people with dementia in general hospital settings, especially from the perspective of the person (Miller et al., 2016; Pecanac et al., 2018). Little attention has been paid to the understandings people with dementia and their families have of the caregiving situation when the person is admitted to hospital, how this may shape perspectives on decision-making, or how these perspectives intersect with those of hospital staff. Most notably, as highlighted by two recent systematic reviews, very little is known about how hospitalized people living with dementia are engaged in understanding and making decisions about their care, and no previous studies have explored their experiences of decision-making (Miller et al., 2016; Pecanac et al., 2018).

Understanding how hospital-based decisions are made, and including the perspectives of people living with dementia, is particularly important because for many people with dementia, a general hospital admission is a “determining event,” resulting in significant care package alterations such as care home admissions or substantial increases in care needs (Fogg et al., 2018). Existing studies focus on end-of-life medical decisions (such as the use of artificial hydration or ventilation), where involvement of people with dementia may be shaped by physical rather than cognitive ill health, excluding decision-making for people with longer-term care needs and family input.

The aims of this study were to explore how the care needs of hospitalized people with dementia are understood by the person, their family, and hospital staff, and how these perspectives intersect to inform decision-making about the person’s care needs.

## Design and Methods

### Data Collection

Data were collected from people living with dementia, their families, and staff on two care-of-older-people hospital wards in northern England. Data collection took place over two 7- to 9-month periods between 2011 and 2013. Ethnographic data collection (participant observations, informal conversations, and in-depth interviews) were used to explore knowledge exchanges and decision-making within dementia care triads. Multiple data collection methods were used to maximize the involvement of people with dementia, whose perspectives have been excluded from previous studies, alongside the other arms of the care triad (families and staff). The use of observations, conversations, and repeated visits enabled the development of relationships with people living with dementia and tailoring of data collection to each person’s communication abilities. This was particularly important in facilitating the inclusion of people with limited verbal communication whose often marginalized perspectives would have been excluded by other methodological approaches. These methods also enabled detailed exploration of interactions between members of the care triad, including longitudinal explorations of how knowledge exchanges and decision-making developed over time.

#### Observational data collection

**Figure 1.**
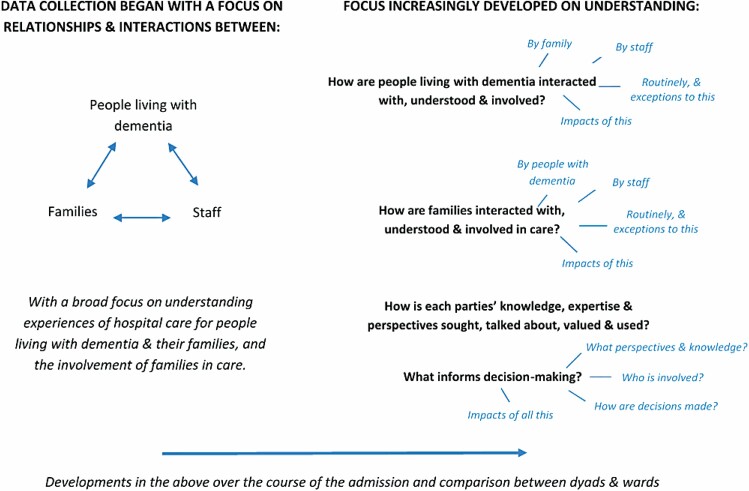
Illustrative observational guide. *Note*: In accordance with the ethnographic, constant comparative approach taken, the focus of the observational guide evolved as the focus of data collection and analysis developed.

Data collection commenced with general observations to develop familiarity with ward routines, environments, and staff. These were followed by in-depth ethnographic case studies (O’Rian, 2009), involving participant observations, conversations during observations and in-depth interviews, with 12 paired dyads of people with dementia and their families (six per site). A range of staff involved with each dyad were observed and interviewed to gather a breadth of experiences. An observational guide (Figure 1) was developed from the general observations, literature review, and emerging analysis, guiding observational attention toward interactions within care triads involving knowledge exchanges and decision-making. Observations focused on situations where knowledge exchanges and decision-making occurred (e.g., ward rounds, team and family meetings, visiting times) in various spaces (e.g., communal areas, meeting rooms, and bed areas).

Four hundred hours of observation were conducted: 190 hr at Site 1 and 210 hr at Site 2. Observations were typically 2–4 hr long (range 0.5–6 hr depending on the activity observed), including mornings (from 8 a.m.), evenings (until 9 p.m.), and weekends. Fieldnotes were handwritten during observations, or shortly afterwards, and typed into fuller versions later, including descriptions of knowledge exchanges and decision-making in hospital records. To avoid depersonalizing any participants, and to reflect the relationships developed with them during data collection, all participants were given pseudonyms rather than being described solely as carers, patients, or staff.

#### Informal conversations and in-depth interviews

Informal discussions and in-depth, semistructured interviews enabled further exploration of knowledge exchanges and decision-making. Informal discussions, undertaken during observations and recorded via audiotape or fieldnotes, occurred regularly with all participants, facilitating the inclusion of people with dementia who were unable to undertake in-depth interviews due to acute illness or confusion. Forty-six in-depth interviews were undertaken with 23 staff, 11 family members (one declined), and four people with dementia. Postdischarge follow-up interviews occurred with eight family members and two people with dementia (the remainder unavailable due to further illness or bereavement). Interview topic guides were developed for each participant group (see Supplementary Material).

Ward-based interviews occurred in private spaces, or occasionally by bedsides due to mobility problems or room unavailability. Postdischarge interviews usually occurred in the person’s residence. Interviews were audiorecorded and transcribed verbatim, varying from 30 min to 1.5 hr in length, depending on the participant’s conversational ability and preferences.

All data were collected by RK, a PhD student who was also an experienced mental health nurse, for her doctoral thesis. Her influence on the data collection was explored via a reflexive diary, as described in Kelley (2017).

### Sampling

The study took place on two care-of-older-people acute hospital wards in two cities: a 24-bed general hospital ward and an 18-bed rehabilitation ward. Theoretical sampling, informed by the emerging analysis, focused on including a diverse range of patient–family dyads (e.g., a range of caregiver relationships, dementia severity, and physical complaints), and staff with varying professional backgrounds, experience, and training.

**Figure 2.**
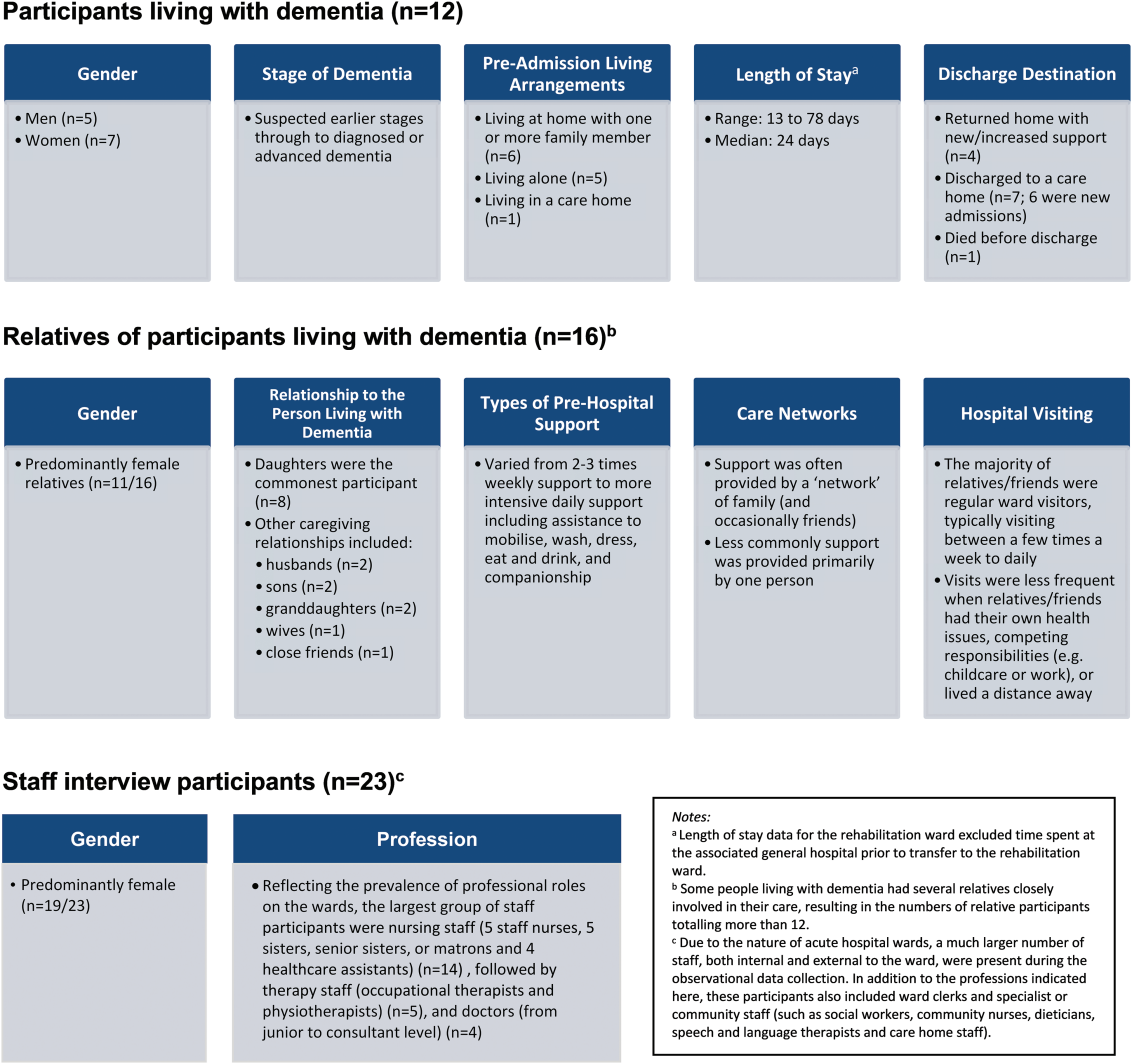
Characteristics of participants.

People living with dementia (*n* = 12) and their families (*n* = 16) were eligible for inclusion if the person had suspected or diagnosed dementia, at least one family member or friend involved in their care, an admission expected to last at least 7 days, and communicated predominantly in English. All ward staff (except agency staff and students) were eligible for inclusion. Staff participants included nurses, doctors, health care assistants, physiotherapists, occupational therapists, and therapy assistants. Further details of the patient–family dyads and staff participants are provided in Figure 2.

### Recruitment and Consent

The general observations were advertised via discussions and posters with verbal consent sought. Written consent was provided by each patient–family dyad and for staff interviews. Staff identified patient–family dyads from their knowledge of each person’s cognition and cues suggesting dementia (e.g., “cognitive impairment,” “memory problems”) in hospital records. Staff approached participants before direct approaches were made. Staff interviewees were approached directly from the researcher’s knowledge of their involvement with each dyad.

Care was taken to explain the study understandably to enable people with dementia to make decisions about taking part wherever possible. Capacity was assessed during these conversations, with personal consultees’ advice sought for people who lacked capacity (Mental Capacity Act, 2005). Ongoing willingness of people with dementia to participate was ascertained verbally and by monitoring for signs of unwillingness to continue (e.g., anxiety/withdrawn behavior in the researcher’s presence). Ethical approval was provided by Leeds-Bradford Research Ethics Committee (ref. 10/H1302/49).

### Data Analysis

Data collection and analysis were informed by a constant comparative approach (Charmaz, 2014). Comparison throughout the analysis (across participants, settings, data sources, and time points) was used to identify patterns and variations within the data (Boeije, 2002). Analysis was undertaken chronologically on each case study in turn, enabling integration of fieldnotes and interviews at the point of analysis and comparison between and within cases. For example, comparisons of knowledge exchanges between and within care triads led to development of the theme “partial perspectives,” exploring the different ways in which people with dementia were understood.

Data collection and analysis formed an iterative cycle with emerging analysis directing subsequent data collection and sampling decisions, enabling the refinement of emerging analytic ideas. Analysis began by reading the fieldnotes and interviews chronologically to develop familiarity with the data and identify recurring patterns, with initial coding ideas noted. This was followed by “open-coding” (using Atlas.ti software), where fieldnotes and interviews were coded “line-by-line,” with a focus on identifying patterns (and variations) in relation to knowledge exchanges and decision-making. Emerging ideas were noted in analytic memos. Key codes and analytic ideas were pursued via increasingly focused coding and data collection, before the amalgamation and elevation of key codes into categories and themes. For example, “partial perspectives” incorporates the categories “between” and “within” group differences and codes exploring why these differences arose. Analytic memo-writing throughout the analysis fostered development of the final analytic account.

## Results

### The Different Types of Knowledge Held by Families, Staff, and People With Dementia

Broadly speaking, all of the individuals involved (people living with dementia, families, and staff) aimed to establish or convey how the person was prior to admission, how they were now, and how they were likely to be in the future—information that determined much of their care in hospital and after discharge. Although there was overlap in the knowledge each group used to build a picture of the person, their knowledge was typically partial, with the potential for divergence, between and within groups, in how the person and their care needs were understood.

### Staff Perceptions of the Person

The primary purpose of the wards, and thus a key focus of knowledge exchanges for staff, was to assess and address predominantly physical care needs. There was thus an emphasis from admission onwards on generating knowledge about each person’s physical (including cognitive) health, function, and risk—to determine how these could be improved to a point where the person could be safely discharged:

Fieldnotes Site 1: Orla (a member of therapy staff*) makes the ward’s first contact with Mavis’s daughter by telephone, explaining “I’ve just seen your mum this morning and wanted to check a couple of things.” She questions Mavis’s usual functioning; for example, confirming she lives in a “warden controlled ground floor flat,” has “pull cords” and “a stick.” She confirms “You do the shopping and housework?,” asking “Are you happy continuing on with that?” She repeats “Goes to memory clinic” and confirms “Does frozen microwave meals” and “Prepares her own food then?” The conversation finishes soon afterwards with “That’s brilliant. Gives us a clear picture of exactly how she was before.”

*“Therapy staff” denotes an occupational therapist, physiotherapist, or therapy assistant.

Organizationally, this focus on physical health, function, and risk was apparent in hospital policies, information posters, and routinized paperwork (e.g., assessment tools and referral forms) and was similarly emphasized in written records and staff discussions. Staff referred to a culture of accountability where they could be held personally responsible for failures to assess physical function and risk adequately, resulting in risk-related knowledge featuring prominently in documentation, discussions, and decision-making:

Interview Site 1, Sophie (therapy staff): If it was only a week ago, you’ve discharged that person, you’ve said they were safe to go home, and they’re back in hospital they’re gonna be asking questions on why they went home in the first place. If you’ve documented clearly that you have registered they are a high falls risk, you’ve had this conversation, you’ve put this in place … there’s less likelihood of it coming back on you as being an unsafe decision.

The attention afforded to physical and cognitive function and risk meant people could be largely understood in these terms. Failure to attend to other aspects of the person—such as their usual routines, care preferences, and social or emotional needs—had fewer ramifications for staff and was less prioritized organizationally. Unlike the discussions and systems used to generate knowledge about physical function and risk, neither ward routinely used conversations, tools, or paperwork for inserting knowledge about an individual’s care preferences and routines into care planning or decision-making. This is not to say that this knowledge was not given any value—attempts were made to use paperwork to collect personal knowledge from families of people living with dementia at both sites—but the use of this information to inform care delivery was not routine:

Interview Site 2, Colin (doctor): As you know we’ve recently introduced the Know Who I Am document … The problem is that, even on my ward where I feel quite passionate about it … it’s still not being used routinely.

Although introducing this document indicated that personal knowledge was valued, understanding physical and cognitive function took priority in the busy and time-limited ward routines. In addition, many staff had not received dementia training explaining how knowledge of someone’s usual routines, behaviors, communication habits, preferences, and life histories could enhance care provision. Staff with this expertise more often sought and included personal knowledge in the picture of the person being created on the ward and the decisions made about them. For example, they were more likely to balance concerns about risk and function against the person’s well-being and preferences:

Fieldnotes Site 2: The team discuss a lady with dementia who is being discharged to a care home. “Does she know that?” asks Frank (a senior, experienced doctor). Another staff member says she is vulnerable at home and that the family are “All in agreement.” “Is she happy with that?” asks Frank.

Support from senior staff for seeking personal knowledge was also influential. At Site 2, the value and use of personal knowledge were encouraged by senior staff, creating a greater expectation that this information would be sought from patients and families, which filtered down to many, although not quite all, staff:

Interview Site 2, Emma (therapy staff): It all feeds down from the consultants really … they are always keen for collateral [history from families].

Interview Site 2, Taj (junior doctor): How is it that knowing my patient used to play golf in the sixties will help me manage him, manage his dementia … How is that relevant to a doctor who is very busy?

### Families’ Perceptions of the Person

Although families could share a focus on physical function and risk, their knowledge of the person came largely from before the hospital admission and so could differ from the views of staff. Families’ understanding of their relative’s well-being in hospital relied on what could be understood from conversations with their relative or staff during visits, the limited bedside notes, and potentially unreliable judgments from their relative’s often minimal activity during visits:

Interview Site 1, Jenny (daughter): It’s guess work because I don’t know … they are not saying to me “Your Dad’s done this today.”

Interview Site 2, Debbie (daughter): When my dad’s in hospital … and he’s taken out of bed and into a chair, we don’t really see what he’s capable of … we don’t really know how much help he needs … They never explained as to what he can do.

Varying degrees of knowledge exchange between staff and families meant some families received limited access to the knowledge staff held about their relative. As a result, some families felt well informed and consulted with, while others felt excluded from decision-making and information about their relative’s care needs:

Interview Site 2, Jeff (husband): It was very helpful … he [doctor] was explaining, as much as he knew, about the condition … at least we were in the picture … you could start planning from then on.

Interview Site 2, Lucy (daughter): We weren’t told about it [the care planning meeting]

Varying levels of engagement with families also resulted in some missed opportunities to understand families’ perspectives on the person, including biographical knowledge of the person’s life history, key relationships and usual behaviors, how closely their current health reflected their usual state, and expert caregiving knowledge. It was possible for staff to hold some of this knowledge if they had cared for the person previously or sought this knowledge from families. However, even when sought, this knowledge formed a small part of an expanse of information focused on other areas which, coupled with busy ward routines, meant it was not always used in practice:

Interview Site 2, Debbie (daughter): We filled it in [document requesting personal knowledge] but ... it didn’t seem as though it was used.

Interview Site 1, Natalie (nurse): How many people actually get time to sit down and read that [personal knowledge document]?

### Perceptions Held by People Living With Dementia of Their Situation

The perspectives of people living with dementia on their current and future care needs could differ from those of families and staff. For example, it was not uncommon for people living with dementia to be unsure of why they were in hospital:

Fieldnotes Site 2: David says he can’t remember why he is in hospital, saying someone brought him here and “Here he is.” I comment that he looks like he’s been in the wars, indicating towards his bruised, cut hands. He agrees, rubbing his hands uncertainly.

People with dementia could be unaware and thereby unconcerned by risks (such as the fall that had occurred above) that concerned staff or families. The person’s views of their current abilities could also differ, with some people believing that they could undertake activities that others felt or knew they could not:

Interview Site 2, Emma (therapy staff): People say “oh yes, I was walking to the shops last week” when actually they have been bed bound for a few months.

However, dementia was not the only factor potentially constraining peoples’ understanding of their current situation and future needs. People with dementia were not always privy to information staff held about them or involved in discussions among staff or families about their future care. Although these exclusions could be made with good intentions, such as to enable frank discussions without causing distress, there were instances where people with dementia were aware that they were being excluded from conversations about their future lives:

Fieldnotes, Site 1: Joan says her daughter told her they are having a meeting this week to decide where she is going to go, as if she may be going into care. I ask if anyone has asked her where she wants to go. She says “No.” She isn’t invited to the meeting.

Failure to involve people with awareness of their situation in conversations about their care—either at all, or in ways that catered for their understanding and emotional needs—could result in distress and missed opportunities to reach a more shared understanding. In contrast, some staff, typically those with more experience or dementia expertise, spoke of the value and potential for including people with dementia in discussions about their careː

Interview Site 1, Natalie [nurse with dementia expertise]: Everybody tends to talk about them and around them [people with dementia] rather than address the issue with the person themselves … I’d one lady who said, “I realise I’ve huge risks if I go home” … “if I can’t manage this time I will then go into placement” … and they all skirted around her really at first … they’d been messing about for weeks. And you think gosh, why didn’t somebody just ask her in the first place.

These examples illustrate how some people with dementia were wrongly assumed to be unable to participate in discussions about their future care. They demonstrate the importance of involving people with dementia in conversations and decisions wherever possible, in ways that maximize their understanding and participation; in some instances, people were involved in discussions without information being conveyed understandably:

Interview Site 1, Doreen (daughter): She [the anaesthetist] was talking to my mum and I kept thinking she’s not even understanding what you’re talking about. And she must have known, cause it’s on her notes, you know, dementia … I thought well she’ll ask me surely, but no … “Do you want an epidural?” … Me mother doesn’t even know what an epidural is, she hadn’t a clue.

Discussions were not always sufficiently sensitive to the person’s communication needs, with the above example also illustrating failure to draw on family to help translate information and maximize understanding. Attempts to involve people could result in distress if the proceedings were unclear or it became apparent the discussion concerned contentious decisions. For example, Ailsa found her inclusion in a meeting with staff regarding her impending transfer to a care home highly distressing due to her inability to follow the discussion around this unwanted transfer:

Fieldnotes Site 1: Two staff members from Ailsa’s new care home visit the ward to assess her. One tries to explain where they are from. Ailsa begins sobbing, asking to go home. The lady tries to comfort her but speaks too quietly for Ailsa to hear [she is deaf]. Ailsa cries repeatedly during the 20-minute discussion, asking to go home, where her children are, and if they are ok.

Collectively, the above examples illustrate regular failures to involve people with dementia who were able to clearly voice their opinions in discussions and decisions about their care, either at all or in ways they could understand.

### Partial Perspectives Within Groups

Partial and inconsistent perspectives were possible within, as well as between, groups. For example, family members could have differing relationships, contact, or caregiving input with relatives with dementia, meaning they could also hold inconsistent views of the person. This could lead to staff being given conflicting views on the person’s capabilities and needs, depending on which family member they spoke to:

Interview Site 2, Emma (therapy staff): One day you see one family member who tells you a long story about what they think should happen! And then the next day you’ve got the opposite!

Family members did not always communicate or agree on what postdischarge care would be best for their relative. Staff then had to identify these inconsistent views before attempting to negotiate a shared understanding of the person and their future care needs:

Interview Site 2, Rita (doctor): There are often disagreements about where people should go … There’s been disagreements like “Oh mum always wanted to be at home, I can’t believe you want to put her in a care home” and the other family going “but we’re reaching the end of the tether.”

The scenarios under which different professions came into contact with patients could also lead to partial or inconsistent understandings between staff members. On both wards, the staff teams consisted of a mix of disciplines with differing degrees of permanency—nurses and health care assistants were attached to the ward whereas doctors, therapy staff, and social workers were typically allocated amounts of time to the ward or to specific patients alongside other duties elsewhere. As a result, while doctors and therapy staff often saw patients at fixed times or undertaking specific tasks, nursing staff supported patients across the day undertaking a range of daily activities, leading to potential for the same person to be viewed quite differently:

Interview Site 2, Emma (therapy staff): We can disagree! … physio and doctors … with the nursing staff because we don’t ever see the patients as the nurses do! … We don’t have to give them help with the general day-to-day things that they need to do to be able to go home.

Discrepant perspectives between staff, sometimes but not always according to discipline, could also result from inaccuracies or inconsistencies from hospital records or the person—including difficulties gaining an accurate picture of people in unfamiliar hospital surroundings who were more confused than usual:

Site 1, Sophie (therapy staff): You’re not getting a clear picture with people with dementia. They function generally so much better in their own environment, so taking them out of their own environment, they’re bound to have more issues than they would at home. And it’s being able to try and judge what’s the environment and what’s their actual cognitive state, and that’s quite difficult sometimes.

### Assimilating Partial Perspectives to Reach Decisions

Recognizing and integrating partial perspectives was an essential but often challenging component of discharge decision-making, particularly when these perspectives diverged. There were times where families, staff, and people with dementia agreed on the best course of action, but there were also occasions where opinions on appropriate discharge plans diverged between or within groups:

Interview Site 2, Emma (therapy staff): Often the doctors will say, right so this person’s doing much better … shall we aim to get them back to the residential home … or aim to get them home by the end of the week? And then they [nurses], or I’ll say, actually this person hasn’t walked since they’ve been in here! They can’t stand up! So we are not going to get them home! … they often get a very different picture to us ... we have to try and put together a picture just between us, never mind family!

Discussions between parties were usually the means through which differing perspectives were identified and attempts made to assimilate them. Multiple, skilled conversations could be required to identify and negotiate strongly divergent views or to make more challenging decisions. Difficulties thereby arose if staff had not begun seeking the views of families or patients early into the admission, with delayed or extended care planning discussions regularly resulting in longer lengths of stay for people with dementia amid mounting organizational pressures to discharge:

Interview Site 2, Colin (doctor): It’s a very difficult decision for people … It takes a bit of time, and you do see people oscillating between what they want ... others it’s very clear cut … it’s hard when you receive an email every day from managers saying beds are tight.

Attempting to assimilate so many different perspectives could lead to some having more influence than others. Perceptions of risk could be particularly powerful in shaping decision-making:

Interview Site 1, Sophie (therapy staff)ː Some patients with dementia don’t necessarily recognise when they’re putting themselves at risk … they don’t understand why you’re not letting them go home … it depends completely on the professionals involved on whether they go home again with the risk that they’re gonna come straight back in or we put our foot down and say “right, they don’t have capacity.”

Fieldnotes Site 1: Nadia (a nurse) says about Ailsa “I hope she goes home” adding she is “so upset, she just wants to go home.” Soon afterwards Paula (one of the therapy staff) joins in, saying “She’s not safe at home” adding “it’s a relief she’s going to placement.”

These quotes reveal the complexity and dilemmas of balancing perceptions of risk against the person’s preferences and emotional well-being, and the significance that decisions made in hospital could have for people with dementia and their families. Decisions to place someone in care, especially against their will, were particularly emotive examples of this complexity:

Interview Site 1, Natalie (nurse with dementia expertise): We get a lot of families coming to us saying “Oh gosh, they really can’t go home” because they’re so worried about the risks, rather than seeing the broader picture, where would they be more happy? And sometimes the risks outweigh the happiness.

Interview Site 1, Beth (senior nurse): [speaking about Ailsa going into care against her will] It’s quite a relaxed hospital environment and it’s been quite distressing her, so I can imagine it’s gonna be the same in a residential home … for her safety it’s a good thing, for her mind, it doesn’t sit well … it’s not nice to think that she may not see her son ever again.

Such dilemmas emphasized how some parties’ concerns could lead to others’ perspectives being unintentionally overlooked, particularly those in less powerful positions—typically patients or sometimes junior staff. In Ailsa’s albeit unusual example, discharge to a care home meant that she would never see her housebound son again. This decision was not taken lightly, occurring after extensive discussions among professionals and her family about the significant risks of her returning home, but without consideration of her primary concern, retaining a connection with her family. Her escalating distress at this decision was heard throughout the ward by her day of discharge:

Fieldnotes Site 1: On entering the ward I hear Ailsa sobbing—her eyes are extremely red. She repeatedly shouts “I want to go home.” One of the nurses tells me Ailsa has been “Really upset … She knows what’s happening, that she doesn’t want.”

The above examples highlight the potential for conflicting views on discharge arrangements for people with dementia, including discharge plans that failed to incorporate, or were explicitly against, the person’s wishes. In such cases, judgments of the person’s capacity could determine the extent of their influence over decisions about their care. Several staff gave examples of discharge arrangements, usually care home admissions, that were made against the wishes of people who lacked capacity. When someone’s capacity was questioned, decision-making power could sway strongly towards staff and families. Decisions were then made in what was considered to be the person’s best interests, which could differ from their expressed wishes, especially given the potential for staff and family perspectives to differ from the person’s:

Interview Site 2, Emma (therapy staff): Yes! That happens quite a lot [people with dementia and families disagreeing over care home transfers]. So then you delve deep into things, looking at capacity … Quite a high proportion of people who come into us from home and then go to a care home … their family wants a care home but the patient said they don’t … what family want for a patient has a huge influence over what happens I think some of the time.

While staff and family perspectives could strongly influence decision-making, the bargaining positioning of people with dementia was much more variable, dependent on their ability to assert their views, their degree of involvement in decision-making forums, and whether they were deemed to have capacity to make decisions about their future care. Assessments of capacity could, therefore, be a crucial determinant of the person’s involvement in decision-making. The quality of capacity assessments were, however, sometimes questioned—particularly when judgments were made at one time point by someone (e.g., from social care) who had never met the person before:

Interview Site 1, Beth (senior nurse): It’s on the day and who’s doing it really … it can be quite, not subjective as such, but I think a lot of it is, it tends to be the people you think won’t have capacity have, and the ones you think won’t … On more than one occasion I’ve been absolutely amazed by the decision … it’s people who spend five minutes with patients who make these decisions …. staff who spend seven hours a day ... with that person aren’t able to make assessments on capacity.

Although staff often spoke of the need to involve people with dementia in decision-making, in practice, their involvement was very variable. Some people’s voices were largely or entirely absent, even if they were able to voice an opinion, especially if they were judged to lack capacity:

Interview Site 1, Natalie (nurse): People sort of do override over the person and go straight to ask the family … In some of the big MDT meetings that I’ve been in, you’ll say “Shall we get the patient to come and sit in?” And it’s like “Oh no” and you think oh gosh, that’s really not good, everybody’s making these decisions for you ... and the person’s sat in the day room, and they’re not being that involved really.

The absence of people with dementia from some decision-making forums, alongside power imbalances and the potential for families or staff to pursue outcomes unwanted by the person, required careful attention to balancing individuals’ perspectives, needs, and motivations:

Interview Site 2, Colin (doctor): I think one factors that view [the family’s] into the planning, whilst at the same time safeguarding the interests of the individual … ensuring that one is acting in their best interest and that we’re taking every step to ensure that’s the case … it’s a very difficult, stressful, emotional time, you can’t really make these sort of decisions easily … it’s hard.

These findings highlight the many complexities hospital staff face when attempting to facilitate shared decision-making for people living with dementia. Balancing the involvement and perspectives of all parties required time and skill. In particular, careful attention was required to the involvement and priorities of people with dementia, and to ensuring there were not overridden by others, features that were only intermittently present in practice.

## Discussion and Implications

This study identifies the potential for partial and discrepant understandings of the needs of people living with dementia within general hospital caregiving triads. A lack of timely and consistent engagement between staff, families, and people with dementia reduced opportunities to integrate the variety of perspectives held. This could lead to difficulties agreeing discharge arrangements—resulting in longer lengths of stay—and the views of one or more parties being overlooked. Crucially, people with dementia and their perspectives were repeatedly excluded from decisions about their future lives, even when they were able to clearly voice their wishes. People deemed to lack capacity were particularly likely to have their wishes overlooked.

Very little previous attention has been paid to the understandings hospitalized people with dementia have of their caregiving situation, the extent to which their perspectives accord with those held by staff and families, or their involvement in decision-making (Miller et al., 2016; Pecanac et al., 2018). Our findings highlight an important issue: that people with dementia in hospital settings are repeatedly and unnecessarily excluded from knowledge exchanges and decision-making, directly contradicting the primary tenet of person-centered care—involving the person—risking reductions in personhood in an environment where it is already under considerable threat. In contrast, greater shared decision-making is reported in community samples (Miller et al., 2016), although power differentials within caregiving triads and the potential for families or health professionals to control conversational agendas and decision-making may still occur (Smebye et al., 2012). Our findings demonstrate that such power differentials are particularly strongly weighted against hospitalized people with dementia, who are additionally marginalized by acute illness, increased confusion, unfamiliar hospital surroundings, and more widespread exclusion, necessitating careful attention and redressing in clinical practice.

In relation to power differentials, we also draw attention to concerns about capacity assessments for people with dementia, both in terms of their reliability and their potential to disempower those deemed to lack capacity. This is despite repeated assertions that people who cannot execute a decision can still communicate their values (Miller et al., 2016). The quality of decision-making for people who lack capacity, including judgments of capacity itself, has received limited attention elsewhere (Emmett et al., 2013; Laird, 2014), as have concerns around whether a focus on safety during discharge planning detracts consideration away from emotional well-being for people who lack capacity (Laird, 2014).

A second key issue identified in this study is the potential for inconsistent understandings of the needs and wishes of hospitalized people with dementia—a particularly important point because of the extent to which families, and sometimes staff, are relied upon as surrogate decision makers (Pecanac et al., 2018). The potential for divergent perspectives *within* triadic arms identified here unmask the largely unacknowledged power and knowledge differentials that can occur within, as well as between, stakeholder groups. Some causes of partial understandings, such as limited engagement with families or the person, could exist for any patient group. However, gaining an accurate picture of the care needs of people with dementia was beset by many additional complexities, including confusion, communication difficulties, and the effects of ill health and unfamiliar hospital environments on the person’s cognition and abilities. This complexity creates unique challenges in trying to ascertain the current and future care needs of hospitalized people with dementia.

**Figure 3.**
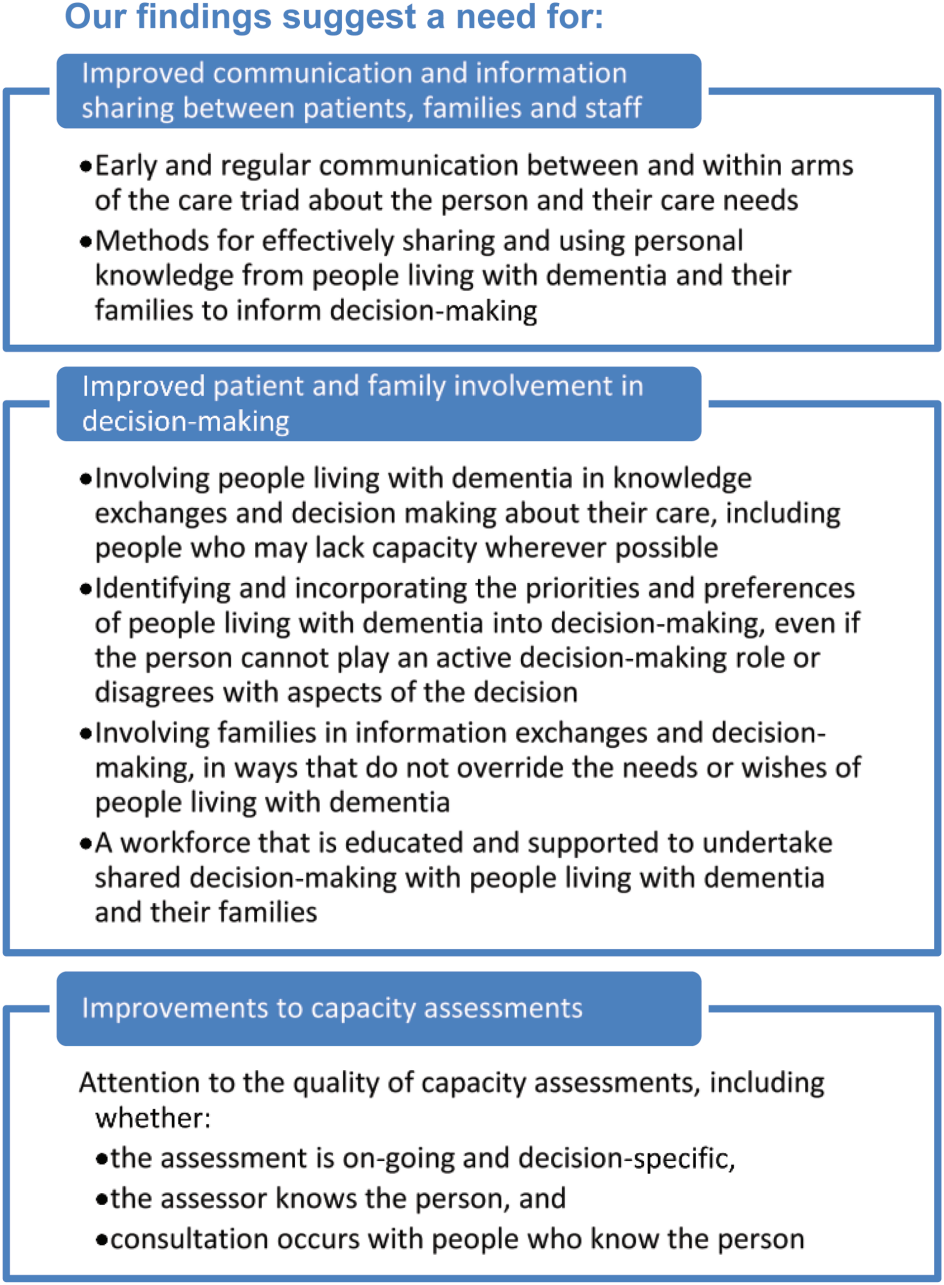
Implications for practice.

As summarized in our implications for practice (Figure 3), the complexity and significance of decisions made in hospital about the future lives of people living with dementia point to a need for improved communication and engagement between and within care triads, with particular attention required to enabling and including the voices of people with dementia, including those without capacity, in discussions *and* decisions about their care. Given recognition that stressed, unsupported families find decision-making particularly difficult (Miller et al., 2016), improving engagement with families and people with dementia seems likely to lead to improved decision-making. However, our findings highlight the need to ensure that families’ input, while clearly important, does not supersede the contributions of people living with dementia to discussions and decisions about their care. In practice, limited opportunities for staff to liaise with each other as well as with patients or families in our study affected identification and integration of individuals’ perspectives. Increasing such opportunities in fast-paced hospital settings is likely to require careful consideration and managerial support.

A recent systematic review (Miller et al., 2016) highlights a need to understand factors that modify decision-making for people with dementia in order to develop future interventions. By identifying modifiable factors that contribute to partial or discrepant understandings, such as insufficient and delayed engagement with families and people living with dementia and a lack of dementia training, our findings also identify ways in which dissonant understandings, and potentially associated discharge delays, could be reduced. Mechanisms proposed to foster community-based shared decision-making, such as involvement continuums, relationship continuity, understanding all viewpoints, and creating space to discuss options (Bunn et al., 2018; Miller et al., 2016), also provide a valuable starting point, but are likely to require adaptions for time-pressured acute hospital settings with large, rotating staff teams. Developing and testing knowledge exchange and decision-making approaches that address the particular complexities of decision-making for hospitalized people with dementia are a valuable focus for future research.

### Strengths and Limitations

Strengths include the length, depth, and multiple methods of data collection, which enabled inclusion of the perspectives of hospitalized people with dementia that are missing from previous research. In addition, collecting data from wards in two hospitals enabled exploration of a broader range of knowledge and decision-making exchanges, patient groups, and ward cultures.

Limitations include predominantly White British participants despite attempts to recruit a diverse sample and the possibility that participants’ experiences were atypical; differences in dementia expertise, discharge planning, and family involvement were reported on other hospital wards. Since collecting these data, national campaigns and audits have focused on improving dementia care in UK general hospitals (e.g., Jones & Gerrard, 2014; Royal College of Psychiatrists, 2019), leading to improvements in dementia training, collection of personal information, and family engagement. Recent reports indicate, however, that patient and family involvement in decision-making remains patchy between and within hospitals (National Federation of Women’s Institutes, 2018; NHS England, 2016), suggesting our findings remain up-to-date.

## Supplementary Material

gnaa216_suppl_Supplementary_Materials
