# Supplementary material for: Knowledge Exchanges and Decision-Making Within Hospital Dementia Care Triads: An Ethnographic Study
Source: Gerontologist. 2021 Feb 18;61(6):954–64. doi: 10.1093/geront/gnaa216 (PMC8521779; doi:10.1093/geront/gnaa216)
Supplement: gnaa216_suppl_Supplementary_Materials [file gnaa216_suppl_supplementary_materials.docx]

**Online supplementary Material for Publication in *The Gerontologist*:**

**Knowledge Exchanges and Decision-Making Within Hospital Dementia Care Triads: An Ethnographic Study**

Rachael Kelley, RMN, PhD^1,2^, Mary Godfrey, M.Social Science^2,†^, John Young, MB, BS, MBA, MSc, FRCP^2^

^1^ Centre for Dementia Research, School of Health and Community Studies, Leeds Beckett University, Leeds, United Kingdom.

^2^ Leeds Institute of Health Sciences, School of Medicine, University of Leeds, Leeds, United Kingdom.

† Mary Godfrey sadly died in September 2020 having made a substantial contribution to this paper prior to its submission.

*Address correspondence to: Rachael Kelley, RMN, PhD, Centre for Dementia Research, School of Health and Community Studies, Leeds Beckett University, LS1 3HE, United Kingdom. E-mail: [r.kelley@leedsbeckett.ac.uk](mailto:r.kelley@leedsbeckett.ac.uk)

**Example interview topic guides**

Separate topic guides were used for the in-depth, semi-structured interviews with people living with dementia, families and staff. The following provides examples of the types of questions that were asked. Questions pertained to information exchanges and decision-making, and also to patient and family experiences and involvement during the hospital admission (the latter reported in a separate paper – [redacted]).

Interview questions were personalised for each individual (to encompass events that had been observed and lines of discussion raised by participants) and evolved over the course of the data collection and concurrent analysis to enable further exploration of emerging avenues of interest. The topic guide for people living with dementia was highly flexible, and was modified according to each person’s understanding and their conversational flow.

**Example interview topic guide – relatives**

**Background information**

- Where and who they live with
- Where the person living with dementia lives
- History of the person’s memory problems
  - *Any help the person needs, who provides it, any strategies that help*

**Admission story**

- Ask for admission story
- How has your relative/friend seemed since they’ve been here?
  - *Any worries? Anything that has been difficult due to their memory problems?*
- Hopes for the outcome of the admission? (Your relative’s? Yours?)

**Talk me through a typical visit to the ward**

- What do you do?
- Conversations with staff?
  - *What do you talk about?*
  - *What sort of information has been shared? (With you? By you? With your relative?)*
- How do you get a sense of how your relative is doing?

**Relative’s involvement during the hospital admission**

- How have you been involved during the admission?
  - *e.g. during assessments, care, sharing information, decision-making?*
  - *Can you talk me through how this happened?*
  - *What impact has your involvement had?*
- How would you like to have been involved?
  - *Actions/roles they would liked to have undertaken?*
  - *Discussions they would have wanted to have?*
  - *What difference would this have made?*
  - *Why didn’t it happen?*

**Example topic guide: person living with dementia**

**Experience of hospital:**

- What is it like here? What happens?
- How do you feel about being here?
- How much choice do you have over what happens here?
  - *Are you involved in deciding what happens? (mention decision specific to the individual)*
  - *What would you like to happen when you leave here?*
  - *Where would you like to go?*

**Relationships and interactions with staff**

- How do you get on with the people here?
- What do the people here know about you?
  - *How well do they know you?*
- What do people here talk to you about?
  - *Do they listen to you? What do you tell them about?*

**Visits, interaction and involvement from relatives**

- Ask about the person’s family
- What does their family do when they visit?
- Who do they talk to?
  - *What about?*
  - *Are you involved?*

Follow up interviews with people living with dementia and families included a greater focus on involvement in decision-making, overall experiences and outcomes of the admission, and what could have been improved.

**Example interview topic guide – staff**

**Patient’s Background**

- What do you know about them?

**Ask for the patient’s admission story**

- Why they were admitted
- Care needed since admission
- Any difficulties with providing that care
- The person’s experience of being in hospital
  - *Involvement in information exchanges and decision-making*
- Anything that has helped

**Involvement of families**

- Describe a typical family visit: What would they do? Who would they talk to? What about?
- Impacts of family visits?
- What sorts of conversations have you had with the person’s family?
  - *What was talked about and why*
- What sort of information has been shared between you?
- How else have family been involved in the person’s hospital stay?
  - *e.g. assessments, care, decision-making*
  - *How did this happen? To what effect?*
- How do you think families of people with dementia should be involved on the ward?
  - *In care? In exchanging information? In decision-making?*
